# Supplementary material for: Large-scale discovery of protein interactions at residue resolution using co-evolution calculated from genomic sequences
Source: Nat Commun. 2021 Mar 2;12:1396. doi: 10.1038/s41467-021-21636-z (PMC7925567; doi:10.1038/s41467-021-21636-z)
Supplement: Supplementary file 13 — Supplementary Data 11 [file 41467_2021_21636_MOESM13_ESM.zip › supplementary_prediction/membrane064436_TATB_RODZ/pwrmsd_membrane064436_TATB_RODZ.pdf]

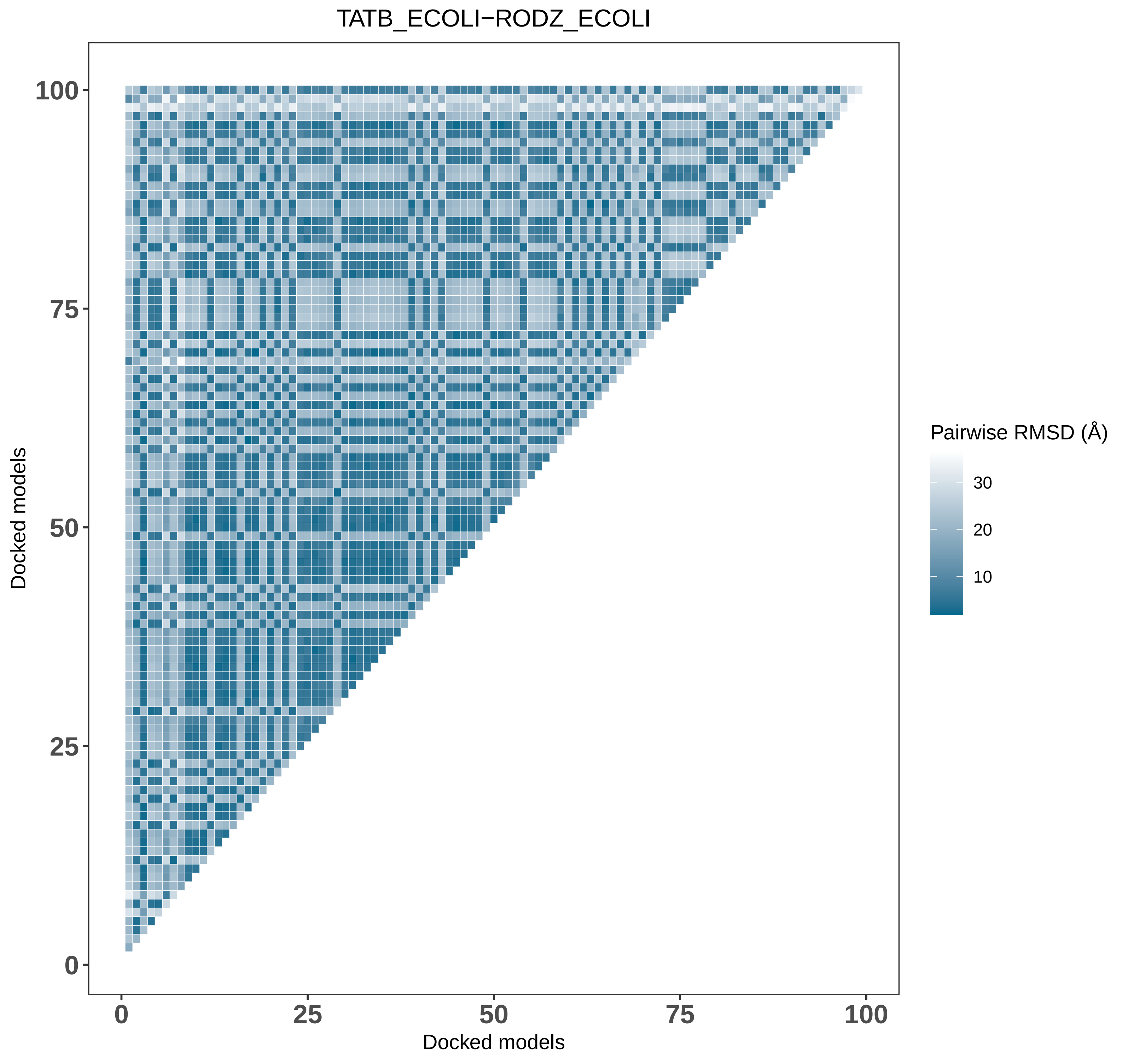

TATB_ECOLI−RODZ_ECOLI
Docked models
Docked models
Pairwise RMSD (Å)
30
20
10
0
25
50
75
100
